# Guidance for the Knowledge and Skills required for Antimicrobial Stewardship Leaders: an update from the Society for Healthcare Epidemiology of America, Infectious Diseases Society of America, Pediatric Infectious Diseases Society, and the Society of Infectious Diseases Pharmacists

**DOI:** 10.1017/ash.2026.10344

**Published:** 2026-05-11

**Authors:** Marisa Holubar, Tanaya Bhowmick, Whitney R. Buckel, Sara E. Cosgrove, Christopher Evans, Thomas M. File, Margaret Fitzpatrick, Elizabeth Leung, Anurag N. Malani, Ana Rios, Jenna Preusker, Priya Nori

**Affiliations:** 1 Stanford University School of Medicine, USA; 2 Stanford Health Care, USA; 3 Rutgers Robert Wood Johnson Medical School, USA; 4 Intermountain Health, USA; 5 Johns Hopkins University School of Medicine, USA; 6 Tennessee Department of Health, USA; 7 Summa Health, USA; 8 Northeast Ohio Medical University, USA; 9 VA Eastern Colorado Health Care System, USA; 10 University of Colorado School of Medicine, USA; 11 Unity Health Toronto, Canada; 12 University of Toronto, Canada; 13 Trinity Health Michigan, USA; 14 Cook Children’s Medical Center, USA; 15 Nebraska Medicine, USA; 16 Nebraska Department of Health and Human Services, USA; 17 Albert Einstein College of Medicine, USA

## Abstract

Antimicrobial stewardship programs (ASPs) optimize antimicrobial use to ensure the best patient outcomes from infections. Since the initial publication of this guidance in 2014, accreditation standards have bolstered antimicrobial stewardship requirements in various healthcare settings, and antibiotic use and resistance data reporting requirements have further increased the scope and resource needs of ASPs. The purpose of this guidance is to outline and update the knowledge and skills necessary to lead ASPs, organized by basic, intermediate, and advanced skills recommended for stewards practicing in acute care, outpatient care, and long-term care settings. This update was developed by a multidisciplinary group of antimicrobial stewardship experts from key organizations and with diverse backgrounds engaged in advancing the practice of antimicrobial stewardship. It does not specifically address healthcare resources, staffing models, reporting structures, or percentage of full-time equivalents needed to conduct this work, which will be addressed by future multi-society publications.

## Introduction

Antimicrobial stewardship refers to coordinated interventions to improve appropriate use of antimicrobials in all healthcare settings (acute care, outpatient care, and long-term care). This is achieved through optimizing antimicrobial regimen, dose, duration of therapy, and route of administration. The objectives of antimicrobial stewardship are to (1) achieve the best clinical outcomes related to antimicrobial use while, (2) minimizing the emergence of antibiotic-resistant organisms, *Clostridioides difficile* infection and other adverse events and (3) reducing excessive costs attributable to suboptimal antimicrobial use.^1^

The Centers for Diseases Control and Prevention endorse antimicrobial stewardship program (ASP) co-leadership from a physician and a pharmacist with subspecialty training in infectious diseases.^2^ Although the in-depth understanding of diagnosis, management, and therapy of infectious diseases afforded by subspecialty training is advantageous, it is not always practical in settings like long-term care, outpatient care, critical access hospitals, and low-and-middle income countries. Fortunately, several high-quality online resources and certificate programs are available for attainment of requisite knowledge and skills.^1–3^ Increasingly, physicians and pharmacists without specialized infectious disease training and infection preventionists are engaged in successful stewardship activities across settings.^4^ Further, expertise beyond clinical infectious diseases, pharmacology, and microbiology is critical to initiate, maintain, and expand an ASP, including fundamentals of quality improvement and change management, effective communication, data presentation, and methods to measure programmatic success.

Recognizing the need for an update to the original guidance,^5^ which continues to be accessed regularly, the Society for Healthcare Epidemiology of America (SHEA) has partnered with the Infectious Diseases Society of America (IDSA), the Pediatric Infectious Diseases Society (PIDS), and the Society of Infectious Diseases Pharmacists (SIDP) to convene a multidisciplinary writing group of experts from academic and non-academic settings and public health departments engaged in advancing antimicrobial stewardship.

The purpose of this update is to reinforce the knowledge and skills outlined in the original document, now organized by basic, intermediate, and advanced skills applicable to acute care, outpatient care, and long-term care settings (Tables 1–3). Core knowledge and skills include understanding and effectively articulating the rationale for antimicrobial stewardship, performing various ASP interventions and activities a program needs for optimal effectiveness, and measuring processes and outcomes associated with an ASP. Collaboration with infection prevention and control (IPC), microbiology, information technology (IT), and institutional leadership to achieve ASP goals are detailed. Finally, skills focused on leadership, program building, and advocacy are enumerated. Collectively, knowledge and skills described in this document intend to help “raise the bar” for ASPs, like the recent guidance document for IPC programs in which Talbot et al. describe “active” (ie, meeting requirements) versus “effective” IPC programs (ie, those which achieve broader patient safety goals and implement proactive rather than reactive measures).^6^ Similarly, this document delineates skills on a spectrum from basic to advanced skills which serve to augment success of ASPs as they evolve over time. It does not specifically address healthcare resources, staffing models, reporting structures, or percentage of full-time equivalents needed to conduct this work, which will be addressed by future multi-society publications.

**Table 1.** Fundamental antimicrobial stewardship Knowledge and Skills—Clinical Infectious Diseases

Knowledge/skill		Acute Care^a^	Out-patient^a^	Long-term care^a^	Basic (B)	Intermediate (I)	Advanced (A)
**Antimicrobials**	Mechanisms of action and spectrum of activity	B, I, A	B, I	B, I	Understand differences in mechanisms of action and spectrum of activity of *commonly used* antimicrobials.	Understand differences in mechanisms of action and spectrum of activity *within* drug classes. Educate frontline prescribers about mechanisms of action and spectrum of activity of *common* antimicrobials.	Educate frontline prescribers about mechanisms of action and spectrum of activity of *less* common antimicrobials (eg those used to treat extensively drug-resistant infections).
	Drug interactions, side effects and adverse events	B, I, A	B, I, A	B, I	Understand common antimicrobial drug interactions and side effects, including adverse reactions that mimic infections.	Perform drug safety audits and root cause analyses when adverse drug events occur.	Develop electronic tools to alert providers to common drug interactions, side effects, and monitoring parameters.
	Allergic reactions	B, I, A	B, I, A	B, I, A	Understand common allergic reactions to antimicrobials and how they can be minimized. Differentiate non-allergic toxicities from true allergies and approaches to antimicrobial allergy de-labeling.	Recognize rarer or complex allergic reactions to antimicrobials (eg, severe cutaneous allergic reactions) and how to escalate care.	Assist in developing and implementing allergy de-labeling programs.
	Antimicrobial pharmaco-kinetics and pharmaco-dynamics (PK/PD)	B, I, A	B	B	Understand PK/PD and their application to appropriate dosing for patient, pathogen and syndrome characteristics. *Priority: physician and pharmacy leads*	Understand extended and continuous infusion, non-standard routes of administration (eg, intraventricular) and dosing in complex populations (eg, renal replacement therapy) *Priority: pharmacy lead*	Understand and apply PK/PD concepts to complex populations and develop corresponding protocols and decision support tools. *Priority: pharmacy lead*
	Therapeutic drug monitoring (TDM)	B, I, A	B	B	Understand *basic* TDM approaches for common antimicrobials (eg, vancomycin) and help develop TDM protocols. *Priority: physician and pharmacy leads*	Understand *complex* antimicrobial TDM approaches (eg, area under curve (AUC) software for vancomycin Bayesian dosing, beta-lactams and antifungals). *Priority: pharmacy lead*	Develop and implement *complex* antimicrobial TDM through electronic decision support. *Priority: pharmacy lead*
	Antimicrobial shortages	B, I, A	B, I	B, I	Assess and respond to antimicrobial shortages and supply chain disruptions affecting antimicrobial use. Develop recommendations for alternative therapeutic approaches during shortages.	Develop communication strategies on drug shortages, time line, and mitigation strategies.	Track and report data pertaining to shortage onset, mitigation, and patient outcomes.
	Antimicrobial resistance	B, I, A	B	B, I	Recognize *common* resistance profiles based on local epidemiology for antimicrobial / organism combinations and distinguish colonization from true infection.	Recognize *less common* resistance profiles based on local epidemiology and design recommendations to optimize treatment.	Recognize viral and fungal resistance profiles, especially in special populations.
		B, I, A	B	B, I	Monitor and educate providers about local resistance patterns. Tailor antimicrobial treatment recommendations to local antibiograms.	Work with Infection Prevention to monitor broad spectrum antimicrobial use and trends in resistance and *C.difficile* infections.	Work with the microbiology laboratory and health departments to monitor resistance at a population level (eg, city/state, outpatient and long-term care populations).
**Clinical Infectious Syndromes**	Research and literature review and evaluation	B, I, A	B, I, A	B, I, A	*Periodically review* literature on the diagnosis and management of infectious diseases, drug trials, and other studies relevant to stewardship.	*Critically appraise* literature on infectious diseases topics relevant to antimicrobial stewardship.	*Apply findings* from recent relevant literature to improve practice at one’s own institution.
	Clinical presentation, diagnosis and appropriate management	B, I, A	B, I, A	B, I	Understand the presentation, diagnosis, and appropriate antimicrobial treatment for *common and/or uncomplicated infections*, such as those of the upper and lower respiratory tract, urinary tract, gastrointestinal tract (including intra-abdominal/pelvic infections and *C. difficile* infections), skin and soft tissue, and bloodstream infections due to the above conditions. Recognize non-infectious mimics of common infections.	Understand the presentation, diagnosis, and appropriate antimicrobial treatment for *complicated and/or severe infections*, such as: central nervous system infections, endovascular infections, bone and joint infections and diabetic foot infections, infections involving prosthetic material and devices, unspecified sepsis, and any syndrome under ‘Basic’ with a more complicated or severe presentation or course.	Understand the presentation, diagnosis, and appropriate antimicrobial treatment for *rare infections* more common in high-risk populations, such as febrile neutropenia, fungal infections, opportunistic infections in immunosuppressed patients, emerging infections and re-emerging vaccine preventable infections.
	Duration of therapy	B, I, A	B, I, A	B, I	Describe the recommended antimicrobial durations for *common and/or uncomplicated* infections with focus on shorter durations (eg, cystitis).	Describe the recommended antimicrobial durations for *complex* infections with focus on shorter durations (eg, complicated urinary tract infections).	Use antimicrobial use data/prescription dashboards to implement interventions to address antibiotic durations and measure impact.
	Antimicrobial prophylaxis	B, I, A	B, I, A	B, I, A	Understand and apply evidence-based recommendations for use of antimicrobials for surgical and device prophylaxis.	Incorporate allergy de-labeling into surgical prophylaxis recommendations. Collaborate with Infection Prevention to incorporate recommendations into prevention bundles.	Develop evidence-based recommendations for antimicrobial and immuno-prophylaxis in high-risk populations (eg, immuno-compromised, neonates)
**Microbiology/Diagnostics Collaboration**	Microbiology lab testing: *indications and ordering*	B, I, A	B, I, A	B, I	Know indications for sending common microbiology tests (eg, culture-based and non-culture-based) on the following specimens/sources: urine, blood, wound, sputum, stool, cerebral spinal fluid.	Develop, disseminate, and implement guidelines for indication-appropriate ordering of common microbiology tests.*	Audit use of cultures and other lab tests to assess for appropriateness.* Implement interventions to improve microbiology lab test ordering* (eg, reflex culture if CSF shows pleocytosis). Track utilization rates over time (eg, blood culture positivity rates). Feedback data to leadership and frontline teams.*
	Microbiology lab testing: *test selection*	B, I, A	B, I, A	B, I, A	Understand differences between culture and non-culture-based microbiology testing (eg, serology, polymerase chain reaction (PCR)).	Develop, disseminate, and implement guidelines to encourage appropriate microbiology test selection (eg, culture vs non-culture-based).*	Implement interventions to encourage appropriate test selection at the time of ordering (eg, clinical decision support at order entry).*
	Microbiology lab testing: *specimen collection*	B, I, A	B, I, A	B, I, A	Describe appropriate methods for obtaining cultures and other relevant samples (eg, PCR) from different body sites.	Develop and disseminate specimen collection guidelines.*	Implement interventions to encourage appropriate specimen collection at the time of ordering (for example using electronic health record) (EHR).*
	Interpretation of microbiology culture results	B, I, A	B, I, A	B, I, A	Distinguish between results representing infection, colonization, and contamination. Recognize errors in test reporting.	Develop strategies to assist clinicians with distinguishing between infection, colonization, and contamination.*	Implement interpretive guidance into electronic decision support systems.*
	Interpretation of non-culture-based microbiology test results (eg, serology, PCR, whole genome sequencing)	B, I, A	B, I, A	B, I, A	Interpret test results based on the testing approach used at the institution. (eg *C. difficile* testing*)*	Determine the optimal *C. difficile* testing strategy for the institution and develop and implement guidelines for interpretation of test results.* Develop interpretive comments in the EHR microbiology report to guide interpretation of testing results in collaboration with the microbiology laboratory.*	Audit use of testing to assess for appropriateness and feedback data to leadership and frontline teams.* (eg, *C. difficile* testing*)* Develop hard-stops for targeted testing orders in the EHR.* Collaborate with the microbiology lab to develop and implement guidelines for appropriate use and interpretation of additional non-culture-based test results (eg, syphilis, multiplex molecular testing).*
	Biomarkers	B, I, A	B, I, A	B, I, A	Understand test characteristics and applications of biomarkers as diagnostic tools. Learn which biomarker testing is in use at the institution.	Assist the laboratory in decisions regarding implementation or de-implementation of biomarker testing.* Develop and disseminate guidance for biomarker test interpretation and associated antimicrobial treatment recommendations.*	Audit use of biomarker testing to assess for appropriate use.* Feedback data to leadership and frontline teams.* Perform real-time interventions with frontline providers to optimize ordering and interpretation of biomarkers.*
	Rapid diagnostics	B, I, A	B, I, A	B	Understand types and utility of rapid diagnostic tests for microbiologic diagnosis. Understand which rapid diagnostic tests are available at the institution (eg, lateral flow for multidrug resident gram-negative pathogens, Strep throat testing).	Develop and disseminate guidance for existing rapid diagnostic test interpretation and associated antimicrobial treatment recommendations.* Evaluate new rapid diagnostic tests for uptake by the microbiology lab (if applicable).	Audit use of rapid testing to assess for appropriateness.* Feedback data to leadership and frontline teams.* Implement nudging strategies to guide frontline providers to appropriate interpretation of rapid diagnostic test results.* Perform real-time interventions with frontline providers to optimize ordering and interpretation of rapid diagnostic tests.*
	Novel diagnostics (eg, metagenomics)	I, A	N/A	N/A	N/A	Recognize and understand indications for novel diagnostics.	Identify which novel diagnostics are preferred in each situation based on the strengths, weaknesses, and diagnostic accuracy of testing available in your microbiology lab or as a send-out test.
	Antibiograms	B, I, A	B, I, A	B, I	Understand Clinical and Laboratory Standards Institute (CLSI) recommendations for constructing antibiograms. Ensure that an annual antibiogram is available. Evaluate information derived from an antibiogram to identify and track resistance, make formulary decisions and prepare optimal clinical pathways and guidelines. Understand the limitations of antibiogram data for individual patient-level treatment decisions.	Understand the impact of CLSI breakpoint changes on microbiology reporting. If clinically appropriate, work with the microbiology lab to develop, interpret, and disseminate antibiograms that are syndrome specific and/or unit specific.	Evaluate and implement electronic tools (eg, within electronic health record, or third party) that use antibiogram data and other chart data to provide patient-specific antibiotic recommendations. Work with the microbiology lab to assess the need for, develop, interpret and disseminate other types of specialized antibiograms (eg, weighted incidence syndromic antibiograms).
	Microbiology lab shortages	B, I, A	B, I	B, I	Be aware of diagnostic testing shortage(s).	In the instance of a diagnostic testing shortage(s), develop mitigation strategies with the microbiology lab and IT department.*	In collaboration with the microbiology lab and IT department, develop a strategy to proactively monitor for future diagnostic testing shortage(s), and plan for potential mitigation strategies.*

Note. *Examples in this category could be considered as diagnostic stewardship activities.

a Column details priority categories for each setting, where applicable. B, basic; I, intermediate; A, advanced. If not listed in this column, that category may not be readily applicable to this setting and should be evaluated locally.

**Table 2.** Fundamental antimicrobial stewardship Knowledge and Skills - Interventions

Knowledge/skill		Acute Care^a^	Out-patient^a^	Long-term care^a^	Basic (B)	Intermediate (I)	Advanced (A)
**Antimicrobial stewardship Interventions**	***Drug-level interventions***						
	Preauthorization	B, I, A	N/A	B	Implement preauthorization of select broad spectrum, new, or expensive antimicrobials within the first 24 hours of therapy or after a specified number of days (depending on staff availability). Train clinicians, including staff pharmacists, on appropriate use of targeted antimicrobials.	Adapt preauthorization policies into the electronic health record.	Conduct regular audits of preauthorization policies to assess adherence and outcomes. Adjust policies based on data and feedback.
	Postprescription prospective audit and feedback	B, I, A	B, I, A	B, I, A	Communicate feedback on antibiotic orders amenable to streamlining (eg, too broad, too long, intravenous to oral switch.^20^) Begin with consequential antibiotics such as fluoroquinolones.	Assess adherence to ASP recommendations. Adjust policies based on data and feedback.	Provide regular feedback data on antibiotic use or prescriptions to frontline prescribers (eg, provider-specific data).
	Intravenous (IV) to oral (PO) conversion	B, I, A	N/A	B, I	Create an IV to PO conversion protocol for *commonly used* antibiotics. Work with frontline pharmacists and nurses to implement the protocol during routine patient care.	Implement an IV to PO conversion protocol for *all applicable* antimicrobials. Incorporate protocol into clinical decision support system to enable automatic, pharmacist-driven IV to PO switch.	Utilize institutional data to track reductions in IV antibiotic use and associated outcomes (eg, cost savings, sustainability metrics) Present results to appropriate institutional committees.
	Antimicrobial dose adjustment for organ dysfunction	B, I, A	B, I	B, I	Establish protocols and/or EHR prompts for adjusting antibiotic doses in patients with renal dysfunction.	Expand protocols to include hepatic dysfunction and a broader range of antibiotics. Embed protocols into the EHR and regularly review and update.	Use data analytics to monitor adherence to dosing protocols, patient outcomes, and antibiotic resistance patterns and report to appropriate institutional committees.
	***Patient-level interventions***						
	Institutional algorithms and guidelines	B, I, A	B, I, A	B, I, A	Create and disseminate clinical guidelines based on national recommendations; include guidance for diagnosis, testing, and infection management and train all relevant staff. Initially focus on up to two guidelines for common infections.	Tailor guidelines/ordersets to institutional needs and resistance patterns. Integrate guidelines into clinical workflows, computerized physician order entry systems, and decision support tools.	Track algorithm and guideline adherence, evaluate changes in antibiotic use trends over time, and report metrics including outcomes (length of stay, readmissions, costs) to quality committees and leadership.
	Implement tools to standardize the process for patient assessment and communication between providers in long-term care facilities.	N/A	N/A	B, I, A	Apply tools such as the Loeb criteria^21^ to create structured checklists.	Perform more detailed assessments of residents using clinical criteria and recognize atypical presentations of infection in elderly patients.	Provide advanced training to staff on clinical assessment and communication strategies.
	Transitions of care	B, I, A	B	B, I, A	Develop policies and tools/processes such as a standardized discharge summary template or after visit summary for outpatients that promote optimal antimicrobial use (eg, include relevant patient education).	Review discharge antibiotic prescriptions for appropriate dose, duration, and bug/drug match. Educate patients about potential side effects. Example: ensure appropriate inpatient/outpatient total duration of therapy for community acquired pneumonia^22^; conduct antimicrobial reconciliation upon admission to long-term care from acute care	Establish a multidisciplinary process during transitions of care and integrate into workflows. Monitor clinical outcomes and patient satisfaction scores. Establish partnerships between hospitals, long-term care facilities, and primary care if discharged to standardize communication.
	Outpatient parenteral antibiotic therapy (OPAT) and/or complex outpatient oral antimicrobial therapy (COPAT) as an extension of ASP (ideally additional resources/staffing provided).	B, I, A	B, I, A	B	Review OPAT/COPAT prescriptions to ensure alignment with clinical guidelines and patient capacity.	Implement a structured OPAT/COPAT review process with standardized templates for patient follow up, monitoring labs, assessing adverse reactions, and plans to track outcomes.	Create a comprehensive, multidisciplinary OPAT/COPAT program with tools integrated in the EHR, protocols for IV to PO transition, and a plan to monitor program metrics.
	Implement tools and strategies for diagnostic stewardship*	B, I, A	B, I	B, I, A	Provide training to clinicians regarding use of common microbiologic tests (eg blood, urine, respiratory tract cultures, stool testing, etc.).	Implement checklists, algorithms and other decision support to guide prescribers toward appropriate test ordering.	Work with Infection Prevention and nursing staff to implement diagnostic stewardship around testing for *C.difficile*, catheter-associated UTI, etc.
	***Institutional-level interventions***						
	Education as a foundational intervention	B, I, A	B, I, A	B, I, A	Identify educational needs based on the setting, audience and compliance with national standards (eg provide at least annual general ASP updates to facility staff electronically).	Tailor educational interventions to specific audiences and settings (eg develop case-based learning modules or a training series tailored to specific discipline or setting).	Evaluate the effectiveness of prior education and combine education with other strategies (eg, antibiotic use monitoring and individualized feedback) for sustained behavior change.
	Formulary management	B, I, A	B, I, A	B	Assess formulary at least annually, making recommendations for streamlining agents and reducing acquisition costs.	Implement therapeutic interchange policies and evaluate newly approved agents.	Align formulary with institutional goals and systemwide ASP efforts including cost savings.
**Informatics**	Utilize electronic health records (EHRs) for antimicrobial and/or diagnostic stewardship functions	B, I, A	B, I	B, I	Assess landscape of existing EHR resources and alerts/flags for stewardship functions.*	Implement and maintain EHR-based stewardship interventions, and update and customize alerts/flags available within the EHR.*	Utilize machine learning, artificial intelligence, and risk scores to optimize management of common syndromes.*
	Assess external antimicrobial stewardship clinical decision support software	B, I, A	B	B	Identify existing decision support programs used at the facility and explore opportunities to improve antimicrobial use.	Evaluate facility needs for external antimicrobial stewardship software/smart phone apps, clinical decision support systems and identify vendors and their products matching facility needs.	Engage local/system vested parties for compatibility and/or implementation and create a business case for acquisition and adoption.
	Develop and implement electronic order sets in EHR	B, I, A	B, I, A	B, I, A	Assess existing order sets and measure utilization among providers.	Update/customize order sets compatible with institutional guidelines.	Update/customize/implement order sets compatible with institutional guidelines and implement targeted education.

Note.^a^ Column details priority categories for each setting, where applicable.

B, basic; I, intermediate; A, advanced. If not listed in this column, that category may not be readily applicable to this setting and should be evaluated locally.

* Examples in this category could be considered as diagnostic stewardship activities.

**Table 3.** Fundamental antimicrobial stewardship Knowledge and Skills – Program Building and Leadership

	Knowledge/skill	Acute care^a^	Out-patient^a^	Long-term care^a^	Basic (B)	Intermediate (I)	Advanced (A)
**Program Building and Leadership**	Maximize effective written and verbal communication skills	B, I, A	B, I, A	B, I	Develop and maintain communication skills to assist in collaboration with a variety of stakeholders Develop specific skills in communicating recommendations to clinicians.	Enhance communication strategies to manage outlier prescribers.	Educate others regarding enhanced communication skills; seek feedback or coaching to advance your communication skills. Optimize communication for collaboration across multiple hospitals and/or health system.
	Conduct strategic planning	B, I, A	B, I	B, I	Develop strategic goals for ASP for 1 year. Align ASP efforts and goals with current institutional strategic initiatives. Reevaluate goals on an annual basis.	Develop strategic goals for ASP for 3–5 years.	Integrate initiatives with other hospital programs Develop strategic goals across multiple institutions and/or health systems.
	Develop proposals for ASP initiation and/or expansion	B, I, A	B, I	B	Develop proposal for ASP initiation using regulatory and accreditation standards and ensure written support from leadership.	Develop proposal for ASP expansion with knowledge of financial and patient safety metrics and benchmarks.	Develop proposal for *health system* ASP expansion using financial and patient safety metrics and benchmarks.
	Develop and implement antimicrobial stewardship education	B, I, A	B, I	B	Develop and present education on antimicrobial stewardship and provider roles and responsibilities.	Assess impact of educational programs (eg, quantify use of ASP-created educational tools).	Develop, implement, and standardize an educational plan across multiple institutions and/or health system.
	Establish microbiology laboratory collaboration	B, I, A	B	B, I	Establish two-way communication with the institution’s microbiology lab leadership Inform the microbiology lab of antimicrobial formulary additions and subtractions so that they can modify susceptibility testing and reporting as needed. Appoint a microbiology representative to the ASP committee, if applicable.	Establish regular (eg, quarterly) standing meeting with microbiology lab leaders to discuss common issues (can be in conjunction with infection control program).	Establish a system that ensures that the ASP and microbiology lab work in tandem to make decisions regarding evaluation, selection, and deployment of microbiology testing strategies.
	Establish pharmacy department collaborations	B, I, A	B, I	B, I	Develop pharmacy leadership support for the ASP and/or specific interventions.	Develop pharmacy leadership support for multiple interventions.	Develop pharmacy support for interventions across multiple hospitals and/or health system.
	Medical-legal implications of antimicrobial stewardship	B, I, A	B, A	B	Understand medical-legal implications of antimicrobial stewardship.	Develop a root cause analysis and reporting process collaboratively with other departments (eg, medication safety, quality) to respond to and prevent antibiotic adverse events like vancomycin-associated acute kidney injury.	Develop processes to communicate and resolve differences with treating teams and/or other stakeholders by working with hospital administration, pharmacy staff, other stakeholders including risk management and legal counsel as necessary.
	Compliance with regulatory standards	B, I, A	B, I, A	B, I, A	Regularly ensure foundational compliance with accreditation standards and regulations.	Develop robust infrastructure for compliance and adhering to standards.	Monitor changes in mandates and respond to state or federal audits. Proactively design systems that exceed current accreditation and regulatory requirements and prepare for future standards.
**Measurement and Analysis**	Measure institutional antimicrobial use	B, I, A	B, I	B	Determine sources of antibiotic use data and relevant inpatient denominator data for the institution (eg, patient days or days present). Understand and determine when to use different standard methods of measuring antimicrobial use and approaches to normalize data including days of therapy (DOT), or length of therapy (LOT).	Identify and apply appropriate antimicrobial use benchmarks within an institution or between similar institutions. Use institutional antibiotic use and benchmark data to identify areas for improvement. Provide feedback antibiotic use data to stakeholders, including prescribers, hospital, unit, service, agent, and/or prescriber/practice.	Develop interactive dashboards to allow on-demand, up-to-date assessments of antimicrobial use by hospital, unit, service, agent, and/or prescriber, including the outpatient setting. Develop, implement and measure the impact of interventions.
	Use data to design antimicrobial stewardship interventions	B, I, A	B, I, A	B, I	Use data to identify simple opportunities for improvement (eg, monthly antibiotic use reports to identify high-use antibiotics). Determine common prescribing indications and design targeted education.	Tailor interventions to specific prescribing patterns or specialized units (eg compare antibiogram data to antibiotic usage rates to develop interventions; use prescription dashboards for acute respiratory infections to drive interventions).	Use data to integrate continuous quality improvement processes that include multifaceted interventions. (eg, Implement a rapid diagnostic stewardship protocol, provider education, and a pharmacist-led real-time feedback program together).
	Report institutional antimicrobial use	B, I, A	B	B	Determine if/what reporting is required for regional or national monitoring and ensure compliance. Understand processes and definitions associated with the regional/national required reporting (eg, NHSN Antimicrobial Use and Resistance (AUR) module). Oversee data submission to regional/national required reporting for the institution. Feedback data to *key* stakeholders.	Use institutional data from regional/national required reporting to understand local antibiotic use and resistance patterns as benchmarked against national data. Use institutional data from the regional/national required reporting to identify areas for improvement Feedback data to *all* stakeholders for example including prescribers, by hospital, unit, service, practice and/or agent.	Develop, implement, and measure the success of interventions to improve antibiotic use based on data from the regional/national reporting.
	Measure adherence to ASP guidelines and interventions	B, I, A	B, I, A	B, I	Measure appropriateness of antibiotic use for common infectious diseases syndromes. Develop an approach to identify/assess adherence to guidelines/ASP interventions.	Measure results of interventions to improve appropriateness of antibiotic use for common infectious diseases syndromes. Feedback data regarding appropriate antibiotic use/adherence to guidelines to stakeholders, including prescribers.	Develop dashboards containing data on inappropriate or non-guideline compliant antibiotic use. Electronically identify inappropriate antibiotic use real-time to facilitate interventions.
	Identify and measure other ASP outcomes	B, I, A	B, I, A	B, I	Understand strengths, limitations and uses of other outcomes including: quality metrics related to antibiotic prescribing (eg, sepsis) and suboptimal antimicrobial use,^23^ patient outcomes (eg, length of hospitalization, mortality, readmissions, revisit rate, rates), proportions of resistant organisms, antibiotic-associated adverse events (eg, hepatotoxicity, nephrotoxicity), time to appropriate therapy, duration of antimicrobial therapy, antimicrobial costs and costs of care.	Identify and measure outcomes that are of most value in assessing the impact of ASP interventions. Feedback select outcomes data to key stakeholders.	Develop dashboards using data from the EHR to measure and report these other relevant outcomes.
	Measure *C. difficile* and multidrug-resistant organism (MDRO) rates	B, I, A	B	B, I	In conjunction with the Infection Control program, track and report facility-onset *C. difficile* and MDRO rates locally and to national/regionally as required (eg, NHSN C. diff LabID Event). Understand relevant definitions of *C diff* infection for regional/national reporting systems.	Use facility data from local/national reporting to identify areas for improvement. Feedback data to stakeholders.	Perform regular evaluation of *C. diff* cases to assess appropriateness of testing and treatment for *C. diff* and prior antibiotic use Develop dashboards containing data on C. diff treatment and rates
	Disseminate institutional ASP data	B, I, A	B, I, A	B, I, A	Report data to institutional stakeholders in a timely, audience-tailored manner that is clear, actionable and promotes engagement (eg, annual reports).	Understand basic approaches to display quality improvement data (eg, run charts).	Understand and use study designs commonly used to evaluate ASP interventions. Write and publish studies and present abstracts at conferences on ASP interventions and outcomes.
**Public Health and Advocacy**	Support and collaborate with state and local public health agencies	B, I, A	B, I	B, I	Identify antimicrobial stewardship and resistance resources and experts at state and local health departments.	Participate in state or local public health meetings. Share relevant public health guidance with ASPs to ensure alignment with best practices. Share antimicrobial use, healthcare-associated infection, and antimicrobial resistance data (eg, through NHSN) with state and local health departments.	Serve in advisory or consultant capacity to state public health healthcare-associated infection/antimicrobial resistance programs. Collaborate with state and local health departments to develop local/regional benchmarks using antimicrobial stewardship and resistance data.
	Support one health initiatives	B, I, A	B, I	B, I	Attend One Health educational activities to understand the connectivity of antimicrobial resistance in human, animal, and environmental health.	Participate regularly in multidisciplinary One Health collaboratives involving public health, veterinary medicine, and others	Engage actively in advocacy and policy efforts to promote stewardship in all human and non-human health sectors according to CDC and World Health Organization (WHO) priorities.
	Work with state and national societies to advance the field of AS and AMR.	B, I, A	B, I, A	B, I, A	Participate in local and national professional societies that promote antimicrobial stewardship efforts.	Serve on state and national professional society committees that promote antimicrobial stewardship.	Work with state and national societies to develop/describe policy proposals related to advancing the field. Use these deliverables for connection with local and national stakeholders, policy makers and legislators for advocacy.

Note. ^a^Column details priority categories for each setting, where applicable.

B, basic; I, intermediate; A, advanced. If not listed in this column, that category may not be readily applicable to this setting and should be evaluated locally.

This document can be used by the individuals overseeing an ASP, by stewardship teams collectively in their evaluation of current and desired program activities, or by trainees pursuing a career in antimicrobial stewardship. It will facilitate evaluation of what additional skills are needed and inform a plan to acquire additional training to effectively direct an ASP. It also serves as a tool to assess educational needs when developing local curricula related to antimicrobial stewardship and as a framework for administrators to determine what knowledge and skills are needed for overseeing an ASP. Finally, this update also includes a supplementary gap assessment tool to augment the tables (Supplementary Table 1).

## Knowledge and skills for implementation, maintenance, and growth of an ASP

The multidisciplinary group of experts delineated key knowledge and skills into the following categories, providing examples and a suggested time frame for acquiring these benchmarks depending on the level of sophistication of a particular ASP. Knowledge and skills may apply to individual program leaders or the stewardship team as a whole (Tables 1–3)

- **Basic—**In this phase, programs are establishing the structure and staffing of an ASP and obtaining necessary administrative support; a leader’s focus is on knowledge, skills, and programmatic elements laid out in the Centers for Disease Control (CDC) Core Elements of antimicrobial stewardship in applicable settings^7^ and The Joint Commission’s standards for antimicrobial stewardship^8^ including antibiotic use and resistance tracking and reporting. ASP leaders must focus on the skills required to *establish* their program.
- **Intermediate**—In this phase, programs must maintain basic programmatic components but have expanded capabilities and resources to grow the program and implement more complex initiatives (eg, electronic health record (EHR)-based interventions, clinical decision support tools, and diagnostic stewardship interventions). ASP leaders must understand the foundation of their ASP and focus on acquiring the knowledge and skills to *advance* their program.
- **Advanced**—In this phase, programs can maintain basic and intermediate programmatic components without significant effort and can secure resources to expand their footprint and impact (eg, develop and disseminate antibiotic use/resistance data dashboards or outpatient/long-term care prescription dashboards, grow staffing and effort to expand into new areas (eg, transitions of care), perform research/produce scholarly output, obtain QI or additional leadership skills, work within a multisite network and participate in multicenter stewardship data collaboratives). ASP leaders at this stage are acquiring knowledge and skills to *extend* their ASP’s reach into new areas.

Table 1 is intended to serve as a framework only, not a checklist for accreditation or compliance, and is not comprehensive but instead provides relevant examples. As a starting point for ASP leaders, we also suggested priority categories (B, I, A) for each general setting (Acute care, Outpatient, and Long-term care). Although all listed skills are important for optimal stewardship, certain skills are prioritized for distinct settings due to the authors’ recognition of resource and staffing constraints in each setting. We also acknowledge that some skills are optional or aspirational and not prioritized if they do not align with programmatic needs and likely vary by setting (eg, consulting for state public health department healthcare-associated infection (HAI)/antimicrobial resistance programs, publication of research findings, National Healthcare Safety Network (NHSN) reporting, collaboration with microbiology laboratories if all testing is sent to tertiary companies etc.). In addition, some skills outlined in Table 1 may be best suited to either the pharmacy lead (eg, expertise in pharmacokinetic/pharmacodynamic principles) or physician lead (eg, effective communication to department heads or hospital executives), while the remaining skills apply to either pharmacy or physician lead, or other champions leading a ASP (eg, expertise in diagnostic stewardship and collaboration with microbiology and infection prevention for implementation). For these reasons, we developed a gap analysis tool to assist ASP leaders identify key areas for improvement depending upon existing and desired program attributes. (Supplementary Table 1). Finally, given the potential for predictive analytics to augment decision support for appropriate treatment of common infectious syndromes like urinary tract infections (UTIs) and pneumonia, all programs should explore local EHR upgrades to enable these functions.^9–12^

## Developing key partnerships with other hospital programs: an important skill

IPC and ASPs are similarly invested in prevention and appropriate diagnosis and treatment of HAIs, like surgical site infections, methicillin-resistant *Staphylococcus aureus* (MRSA) bacteremia, *C.difficile* infections, and others. Tracking performance measures pertaining to HAI rates and antimicrobial resistance apply to both programs. Physicians may have leadership roles in both IPC and ASP and/or report to a common executive leader, such as the chief quality or chief medical officer. Therefore, resources (including financial, technological, and analytic support) may come from the same source and be shared across programs. Moreover, collaboration with the clinical microbiology laboratory is a critical pillar of both IPC and ASP, particularly for diagnostic stewardship for HAIs (eg, ensuring appropriate testing for *C. difficile* infections, UTIs, and bacteremias), which should be considered a priority area for all hospitals.^13^ Effective, multidisciplinary diagnostic stewardship involves providing training to frontline providers and nurses on appropriate urine, blood, respiratory, and/or stool testing based on patient symptoms, and implementing checklists or algorithms on appropriate test ordering and collection with feedback mechanisms to these providers. An example of this in action was the 2024 blood culture bottle shortage requiring expertise in diagnostic stewardship to develop evidence-based algorithms to prioritize resource utilization.^14^ Other key collaborations among IPC, ASP, and microbiology include surveillance for antimicrobial resistance, and mitigation of facility outbreaks. Attainment of skills such as quality improvement and implementation science augment the effectiveness of both programs, which is also contingent upon successful communication, change management, and data analytic skills. Additionally, successful implementation of ASP or IPC interventions across large hospitals, health systems, or outpatient networks requires close partnership with IT for maximal dissemination.

Certain patient outcomes are of critical importance to ASP and hospital leadership like readmission prevention, length of stay reductions, and clinical cure from conditions like bacteremia and sepsis. Programs such as outpatient parenteral antibiotic therapy (OPAT) which are often staffed by infectious diseases specialists who may or may not be part of the ASP help optimize these outcomes. Sepsis is another key growth area for ASPs. New opportunities for de-implementation of interventions, including those related to sepsis regulatory measures, may enhance ASP efforts to curb excess antimicrobial use. An important skill for ASP leaders is surveying the future regulatory landscape, anticipating and adapting to changes, and implementing those changes locally. Establishing relationships with other facilities and organizations via collaboratives, health departments, and/or professional societies can help ASP leaders stay abreast of regulatory changes.

## Barriers to implementation of ASP interventions

Regardless of skill level or setting, all programs face human, structural, or resource barriers to implementation of ASP best practices. These include:

1. Lack of leadership commitment

Building awareness of antibiotic stewardship’s importance requires visible leadership support. We recommend aligning ASP goals with institutional priorities through quality metrics and regulatory compliance (eg, Centers for Medicare and Medicaid Services (CMS) Healthcare Effectiveness Data and Information Set (HEDIS) measures related to appropriate antibiotic prescribing, or reducing 30-day readmissions for pneumonia.^15,16^) We also recommend meeting regularly with Quality and Safety department and preparing data with common executive-level or “C-suite” metrics such as costs, length of stay, and readmissions.

1. Lack of prescriber buy-in to ASP recommendations

Strategies to address this include:

- Providing prescriber education on updated guidelines, potential antibiotic adverse effects, and increased resistance and microbiome disruption.
- Identifying outlier prescribers and providing comparative data and/or feedback using behavioral psychology techniques devices used within the IDSA Stewardship Curriculum.^1,17^
- Establishing clinical champions, including within the infectious diseases division and outlier prescribers, and an escalation process to address outlier prescribers (eg, informing the clinical chief).
- Providing incentives to prescribers, including financial.

1. Limited awareness of ASP strategies among frontline staff

Building awareness, trust, and rapport with frontline staff, including prescribers with varied backgrounds, can occur through regular communications, including face-to-face interactions, and maximum accessibility to local ASP expertise. We recommend serving as an approachable resource and providing support to frontline staff through trainings and tools like quick-reference guides or algorithms. Empower clinical champions to be integrally involved in ASP initiatives.

1. Lack of resources (time, IT-trained personnel, etc.) ^18,19^

Regulatory requirements, such as those for antibiotic use data reporting, can be leveraged for IT support for EHR upgrades. ASPs can also adopt existing EHR builds in place at other facilities using the same EHR system (eg, *C.difficile* diagnostic stewardship alerts), as codes can be shared among IT analysts. Finally, infectious diseases physician or pharmacy expertise can be extended across a growing health system via tele-stewardship services. For staff training and education on appropriate antibiotic use, ASPs can utilize free tools such as online modules and webinars where available.^18,19^ Finally, stewardship efforts can be extended through champions readily engaged in ASP activities, (eg, frontline pharmacy and nursing-driven intravenous to oral conversions).

## Conclusion

ASPs are critical patient safety and healthcare optimization programs with increasing scope, regulatory requirements, and visibility since the original publication of this multi-society guidance document. In addition to outlining and updating the knowledge and skills necessary to lead ASPs, it offers further specificity by delineating basic, intermediate, and advanced skills applicable to acute care, outpatient care, and long-term care settings according to antimicrobial stewardship experts from key organizations and diverse backgrounds engaged in advancing antimicrobial stewardship. Finally, a gap assessment tool is provided to identify aspirational knowledge and skills and help programs develop work plans to meet these goals.

## Supporting information

10.1017/ash.2026.10344.sm001 Holubar et al. supplementary material Holubar et al. supplementary material

## Supplementary material

The supplementary material for this article can be found at https://doi.org/10.1017/ash.2026.10344.

## Review

The document was reviewed by the SHEA Antimicrobial Stewardship Committee, the SHEA Publications Committee, IDSA, PIDS and SIDP, and endorsed by the SHEA Board of Trustees, SIDP Board of Directors, IDSA Board of Directors, and PIDS Board of Directors.
