# Supplementary material for: Guidance for the Knowledge and Skills required for Antimicrobial Stewardship Leaders: an update from the Society for Healthcare Epidemiology of America, Infectious Diseases Society of America, Pediatric Infectious Diseases Society, and the Society of Infectious Diseases Pharmacists
Source: Antimicrob Steward Healthc Epidemiol. 2026 May 11;6(1):e130. doi: 10.1017/ash.2026.10344 (PMC13162062; doi:10.1017/ash.2026.10344)
Supplement: Holubar et al. supplementary material [file S2732494X26103441sup001.docx]

This *gap analysis for knowledge and skills required for antimicrobial stewardship leaders* assesses existing versus needed strategies, processes, technologies, or competencies required for successful leadership of an antimicrobial stewardship program. It is meant to be used as a supplement to the tables in the manuscript, not as a replacement. It should be completed by stewardship leaders, then discussed with leadership to pursue actions needed to meet stewardship goals.

**Table 1a. Fundamental Antimicrobial Stewardship Knowledge & Skills - Clinical Infectious Diseases**

| **Knowledge/Skill** | | **Status (basic, intermediate, or advanced)** | **Desired State (basic, intermediate or advanced)** | **Plan to Address Gap** | **Timeframe for Achievement** | **Resources Needed** |
| --- | --- | --- | --- | --- | --- | --- |
| **Antimicrobials** | Mechanisms of action and spectrum of activity |  |  |  |  |  |
|  | Drug interactions, side effects and adverse events |  |  |  |  |  |
|  | Allergic reactions |  |  |  |  |  |
|  | Antimicrobial pharmaco-kinetics and pharmaco-dynamics (PK/PD) |  |  |  |  |  |
|  | Therapeutic drug monitoring (TDM) |  |  |  |  |  |
|  | Antimicrobial shortages |  |  |  |  |  |
|  | Antimicrobial resistance |  |  |  |  |  |
|  |  |  |  |  |  |  |
| **Clinical** **infectious** **syndromes** | Research and literature review and evaluation |  |  |  |  |  |
|  | Presentation, diagnosis and appropriate management |  |  |  |  |  |
|  | Duration of therapy |  |  |  |  |  |
|  | Antimicrobial prophylaxis |  |  |  |  |  |
| **Microbiology/Diagnostics Collaboration** | Microbiology Lab Testing: Indications and Ordering |  |  |  |  |  |
|  | Microbiology Lab Testing: Test Selection |  |  |  |  |  |
|  | Microbiology Lab Specimen Collection |  |  |  |  |  |
|  | Interpretation of microbiology test results: General |  |  |  |  |  |
|  | Interpretation of non-culture based (e.g., serology, PCR, whole genome sequencing) microbiology test results |  |  |  |  |  |
|  | Rapid Diagnostics |  |  |  |  |  |
|  | Biomarkers |  |  |  |  |  |
|  | Novel Diagnostics (e.g., metagenomics) |  |  |  |  |  |
|  | Antibiograms |  |  |  |  |  |
|  | Microbiology lab shortages |  |  |  |  |  |

**Table 1b. Fundamental Antimicrobial Stewardship Knowledge & Skills – Interventions**

| **Knowledge/Skill** | | | **Status (basic, intermediate, or advanced)** | **Desired State (basic, intermediate or advanced)** | **Plan to address Gap** | **Timeframe for Completion** | **Resources needed** |
| --- | --- | --- | --- | --- | --- | --- | --- |
| **Antimicrobial Stewardship Interventions** |  | ***Drug-level interventions*** | | | | | |
|  | Pre-authorization | |  |  |  |  |  |
|  | Post-prescription prospective audit and feedback | |  |  |  |  |  |
|  | Intravenous (IV) to oral (PO) conversion | |  |  |  |  |  |
|  | Antimicrobial dose adjustment for organ dysfunction | |  |  |  |  |  |
|  |  | ***Patient-level interventions*** | | | | | |
|  | Institutional algorithms and guidelines | |  |  |  |  |  |
|  | Implement tools to standardize the process for patient assessment and communication with providers | |  |  |  |  |  |
|  | Transitions of care | |  |  |  |  |  |
|  | Outpatient parenteral antibiotic therapy (OPAT) and/or complex outpatient oral antimicrobial therapy (COPAT) | |  |  |  |  |  |
|  | Implement tools and strategies for diagnostic stewardship | |  |  |  |  |  |
|  |  | ***Institutional-level interventions*** | | | | | |
|  | Education as a foundational intervention | |  |  |  |  |  |
|  | Formulary management | |  |  |  |  |  |
| **Informatics** | Utilize Electronic Health Records (EHR) for stewardship functions | |  |  |  |  |  |
|  | Assess external antimicrobial stewardship clinical decision support software | |  |  |  |  |  |
|  | Develop and implement electronic order sets in EHR | |  |  |  |  |  |

**Table 1c. Fundamental Antimicrobial Stewardship Knowledge & Skills – Program Building & Leadership**

| **Knowledge/Skill** | | **Status (basic, intermediate, or advanced)** | **Desired State (basic, intermediate or advanced)** | **Plan to address Gap** | **Timeframe for Completion** | **Resources needed** |
| --- | --- | --- | --- | --- | --- | --- |
| **Program Building and Leadership** | Maximize effective written and verbal communication skills |  |  |  |  |  |
|  | Conduct strategic planning |  |  |  |  |  |
|  | Develop proposals for ASP initiation and/or expansion |  |  |  |  |  |
|  | Develop and implement antimicrobial stewardship education |  |  |  |  |  |
|  | Establish microbiology laboratory collaboration |  |  |  |  |  |
|  | Establish pharmacy department collaborations |  |  |  |  |  |
|  | Medical-legal implications of antimicrobial stewardship |  |  |  |  |  |
|  | Compliance with regulatory standards |  |  |  |  |  |
| **Measurement & Analysis** | Measure Institutional Antimicrobial Use |  |  |  |  |  |
|  | Use data to design antimicrobial stewardship interventions |  |  |  |  |  |
|  | Report Institutional Antimicrobial Use |  |  |  |  |  |
|  | Measure Adherence to Stewardship Guidelines and Interventions |  |  |  |  |  |
|  | Identify and Measure Other Stewardship Outcomes |  |  |  |  |  |
|  | Measure *C. difficile* and Multidrug resistant organism (MDRO) rates |  |  |  |  |  |
|  | Measure Institutional Antimicrobial Use |  |  |  |  |  |
| **Public Health & Advocacy** | Support and collaborate with state and local public health agencies |  |  |  |  |  |
|  | Support One Health collaboratives |  |  |  |  |  |
|  | Work with state and national societies to advance the field of AS and AMR. |  |  |  |  |  |
